# Host niche-specific challenges hindering the treatment of polymicrobial infections

**DOI:** 10.1128/jb.00142-25

**Published:** 2025-08-20

**Authors:** Caroline Black, Catherine A. Wakeman

**Affiliations:** 1 Department of Biological Sciences, Texas Tech University 124573 , Lubbock, Texas, USA; Geisel School of Medicine at Dartmouth, Hanover, New Hampshire, USA

**Keywords:** polymicrobial communities, antimicrobial recalcitrance, host infection sites

## Abstract

Antimicrobial recalcitrance is a growing problem in today’s world. Not only are bacteria developing resistance at an alarming pace but the antibiotic discovery pipeline has gone dry, making antimicrobial stewardship essential for preserving the activity of the antibiotics still currently available for use. In addition to resistance, bacteria also display tolerance to certain treatments as they adapt to their body site-specific niche and cooperate with other organisms in polymicrobial communities. Thus, new and existing antibiotics must contend with altered bacterial metabolism, polymicrobial synergy, increased biofilm production, and nutrient-related adaptations present within specific infectious sites. Finally, these treatments must face the challenging process of moving to the infection site and doing their job without causing harm to the patient. This minireview explores the difficulties antimicrobials face when challenging organisms at different body sites, focusing on the niche-specific dynamics present at sites of infection.

## INTRODUCTION

Antimicrobial recalcitrance in pathogenic microbes is a growing problem around the world (1–3). In addition to struggling to keep up with the growing multitude of organisms evolving resistance mechanisms, we must also contend with a variety of other issues that hinder antibiotic effectiveness. For example, polymicrobial infections are now understood to be a major problem in the world of antimicrobial recalcitrance due to the ability of certain microbial communities to work together via polymicrobial synergism. In fact, many infections that are recalcitrant to antimicrobials have been determined to be polymicrobial (4). Polymicrobial infections have been shown to significantly increase the cost of patient treatment and hospital length of stay, costing thousands of dollars for the individual (5). Polymicrobial chronic wounds alone now cost more than 28 billion dollars a year and affect more than five to seven million Americans every year (6, 7). In addition to battling complex communities of microorganisms, we must also determine the best way to prescribe the same antibiotics across multiple body sites with minimal impact on patients. Antibiotic prescription can have negative impacts at sites across the body, including allergic reactions and inhibiting or killing the host microbiome (8, 9). As every patient is unique (different immune responses, hormone levels, and ages), and each body site is different (nutrient composition and stressors), we must adapt our antibiotic prescription methods to most effectively treat the individual and their illness. This minireview focuses on the challenges that hinder effective antimicrobial prescription across the body, emphasizing the importance of accounting for distinct polymicrobial communities and niche-specific stressors to the bacteria that differ at each body site.

## PHARMACOKINETICS AND PHARMACODYNAMICS ARE IMPACTED BY BODY SITE

One of the biggest challenges with prescribing antimicrobials is getting the drug to the appropriate body site without causing harm to the patient. To be deemed safe and effective, antimicrobials must be able to make it through the body without being metabolized too quickly, absorbed in the wrong location, or causing unforeseen adverse effects. This set of potential concerns is referred to as pharmacokinetics (10). Antimicrobials must also be able to bind to the correct receptor at the site of infection (such as the 30S ribosome, DNA gyrase, and peptidoglycan), at the correct concentration, and do minimal or no harm at the infection site. This series of hurdles is referred to as pharmacodynamics (10). Antimicrobial drugs must overcome the challenges of both reaching their specific body site and working effectively once there in order for antimicrobial prescription to be effective (11). Depending on the body site and the path of drug delivery, the antimicrobial may face pH changes, changes in oxygen levels, altered salt concentrations, and many more challenges. Penetration of tissues and organs can also be a huge issue, forcing the implementation of active drug delivery (requiring external intervention), instead of passive drug delivery (a combination of the drug’s natural properties and the body’s physiological processes allows the drug to passively move to the appropriate site) (12). For example, daptomycin should not be used for pulmonary infections as it is inactivated by surfactants found in the lungs (10). However, daptomycin can be effective in urinary tract infections because of how concentrated it becomes within urine (10). Penicillin and metronidazole have displayed penetration issues for bone and joint infections (13). Nitrofurantoin is very effective for urinary tract infections as it concentrates in the urine and its activity is enhanced by an acidic pH but is less effective in tissues where it can only achieve low levels (14). Many antibiotics are divided into concentration-dependent or time-dependent categories (10). Concentration-dependent antibiotics must maintain a certain level of the drug to be effective. Concentration-dependent antibiotics include aminoglycosides, lipopeptides, and fluoroquinolones (10). Time-dependent antibiotics must remain in the site of infection for a minimum length of time to be effective. Time-dependent antibiotics include penicillins, cephalosporins, carbapenems, vancomycin, oxazolidines, and macrolides (10). Time-dependent antibiotics often require lower dosages more frequently, while concentration-dependent antibiotics tend to require larger dosages (10). To eliminate these problems, many drug developers have turned their focus to alternative and improved methods of drug delivery. These include, but are not limited to using nanoparticles, encasing the drugs in polymers, and creating prodrug forms of the antimicrobials, which can be broken down into the drug’s active form via protease or other metabolic activity in the body (15–18).

In addition, there are issues with determining initial dosages and resistance breakpoints, as both *in vitro* and *in vivo* studies are not always truly indicative of what will happen in human infections. *In vitro* antimicrobial susceptibility testing (AST) has many issues, including lack of clinical relevance as standard media is used (typically Cation-Adjusted Mueller–Hinton Broth (CAMHB) or a similar media is used with clinically relevant concentrations of the antibiotic to determine AST results via visible turbidity), no way to account for the immune system’s response to the drug and potential synergy or antagonism that could occur, and complete disregard for the pharmacokinetics and pharmacodynamics of the antimicrobial that are relevant in a human patient but irrelevant in a test tube or 96-well plate (19–22). There have been efforts to implement models that mimic human pharmacokinetics and pharmacodynamics when performing antimicrobial testing; however, these methods lag behind bacteria’s ability to develop recalcitrance mechanisms (23). The move into *in vivo* AST in animal models such as mice has not been without problems either. Calculating the most effective dosage when the dosages vary significantly between the smaller mouse models and much larger human patients is a challenge, as well as differing immune responses and receptor site recognition issues (24, 25). There is also a significant lack of studies bridging *in vitro* and *in vivo* work and then from *in vivo* work to the clinical setting (26, 27). This research gap makes it exceedingly difficult to apply the recalcitrance mechanisms discovered in a research laboratory to the clinical setting. This disconnect between research and clinical application delays improvements in antimicrobial prescription and stewardship, giving the pathogens the upper hand.

## SITE-SPECIFIC STRESSORS AFFECT ANTIBIOTIC EFFICACY

**Fig 1:**
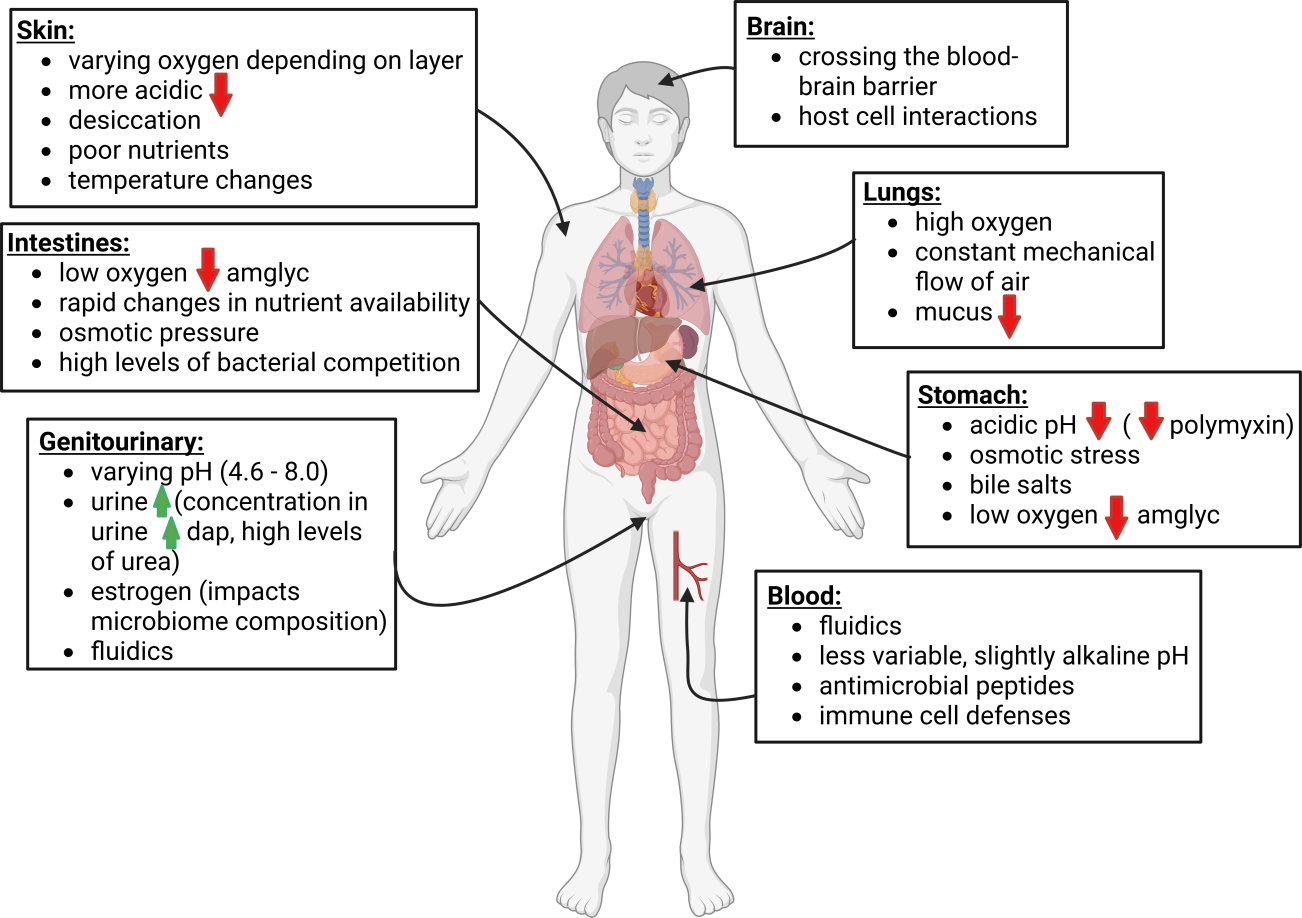
Microbial stressors at different sites across the body. Microbial stressors differ depending on the site of infection. As each body site is different, microorganisms must adapt to that site’s specific stressors to survive and infect. Green up arrows indicate increased susceptibility to antibiotics related to the site-specific stressor. Red down arrows indicate decreased susceptibility to antibiotics related to the site-specific stressor. Abbreviations next to the arrow indicate that the susceptibility change is associated with certain antibiotic(s). When no abbreviation is provided, the change is associated with multiple antibiotics. “dap”—daptomycin and “amglyc”—aminoglycosides.

In addition to every patient being unique, every infection site across the body is different, and therefore, may require altered administration of antimicrobials (Fig. 1). Bacteria can adapt to site-specific stressors, and these physiological adaptations can lead to changes in antimicrobial susceptibility.

Oxygen levels vary across the body, from highly oxygenated sites like the lungs and skin to more poorly oxygenated sites like the intestines. These variances in oxygen levels not only affect what bacteria can grow at different body sites but also affect the physiology of the bacteria present at that body site. Bacterial antimicrobial susceptibilities differ based on whether the environment the bacteria grow in is aerobic or anaerobic (28). Limiting the amount of oxygen available in the environment can significantly decrease an organism’s antimicrobial susceptibility to multiple antibiotics. This has been shown with many organisms, including important clinically relevant pathogens like *Pseudomonas aeruginosa* (which displayed decreased susceptibilities to tobramycin, ciprofloxacin, carbenicillin, ceftazidime, chloramphenicol, and tetracycline in an anoxic environment) (29). This is especially true for aminoglycosides such as gentamicin, which is most effective in aerobic environments as it relies on proton motive force to get into the cell (29). Without aerobic respiration, the proton motive force is altered, and gentamicin is rendered ineffective as it can no longer enter the cell to reach its target (the 30S ribosome). Modulation of anaerobic respiration has also been shown to lead to decreased antimicrobial susceptibility (30). This modulation can be done via small RNAs post-transcription and help regulate reactive oxygen species and homeostasis (30).

pH levels are another important factor that affects an organism’s antimicrobial susceptibility. While body sites such as the bloodstream possess a more constant pH, areas such as the urinary tract, where urine pH can range from 4.6 to 8.0 and varies based on the sex of the patient and their diet, can be difficult to predict without testing (31). pH has been shown to directly affect bacterial phenotypes and antimicrobial susceptibilities (32–35). This has been shown to be especially problematic with uropathogenic bacteria as they have proven to be very adaptable to pH changes and use these changes to their advantage to survive under antibiotic stress, even against antibiotics that concentrate in the urine (34). *Escherichia coli* can become resistant to polymyxin using acid-fitness genes, such as the ones that reduce outer membrane permeability (36). More acidic environments can induce upregulation of biofilm formation in organisms like *P. aeruginosa*, leading to increased antibiotic tolerance (35) as the antibiotic cannot penetrate the biofilm. It can also lead to mutations in efflux pumps, which can also pump more antibiotics out of the cell (35).

Nutrient composition is one of the most variable environmental factors across body sites, as sites such as the intestines have a rich nutrient composition; however, sites such as the skin are considered nutrient-poor for bacterial growth (36). Iron limitation and carbon availability are considered two of the main limiting factors for bacterial growth, and sequestering these nutrient sources away from infectious bacteria is considered a crucial mechanism of the host’s immune system’s defenses.

Iron starvation has been shown to change antimicrobial susceptibilities, as AST results collected in iron-limited media differ from those collected in iron-rich media (37). Iron restriction can induce the production of small colony variants (SCVs) in certain bacterial strains, and these SCVs are often more recalcitrant toward many antibiotics (like aminoglycosides) due to altered metabolism (38). SCVs can also have a competitive advantage for withstanding heme toxicity, indicating that heme-rich niches might also harbor the bacterial strains with inherent antimicrobial recalcitrance (39). Compounds containing iron, like heme, can activate mechanisms of resistance in organisms, such as *Enterococcus faecalis*, containing a heme-dependent catalase (when heme is provided, it can be used in catalase synthesis) that can protect it from reactive oxygen species (40). However, studies have shown that targeting mechanisms of iron homeostasis, thus inducing metabolic stress, can allow us to reinstate susceptibility to antibiotics, such as loss of colistin resistance in *Acinetobacter baumannii* (41).

The carbon sources present in an infection environment can also influence the antimicrobial susceptibility of the organisms growing in that environment. Bacteria can adapt to utilize the carbon sources that become available, as shown in cases such as *P. aeruginosa,* upregulating malonate utilization genes when present in the bloodstream of trauma patients (42). When growing *P. aeruginosa* with malonate versus glycerol as a carbon source, antimicrobial susceptibilities changed. For example, *P. aeruginosa* was less susceptible to aminoglycosides in malonate but less susceptible to fluoroquinolones in glycerol (43). For the malonate media, this is likely due to the inhibition of respiration via malonate, thus altering the proton motive force, inhibiting gentamicin from getting into the cell. Growth of *P. aeruginosa* in malonate also reduced the production of pyoverdine, elastases, and rhamnolipids, while enhancing catalase and pyocyanin production (44). Nutrient sources matter across other species and infection stages as well. *E. faecalis* utilizes purine biosynthesis during early wound colonization as it requires a large amount of purine metabolites during this time (45). However, after the establishment of the initial infection, the MptABCD phosphotransferase system is important for importing mannose and galactose to maintain bacterial persistence within the wound environment, especially when faced with changing carbohydrate availability within the wound site (45). Studies have shown that these could be potential mechanisms to target when developing new antimicrobials. Overall, nutrient availability plays a huge role in determining an organism’s antimicrobial susceptibilities (46).

The presence of other compounds also influences antimicrobial susceptibility. In the urinary tract, urine itself can suppress the mucoid phenotype of organisms like *Klebsiella pneumoniae* (47). Mucoid phenotypes are caused by increased production of polysaccharides like alginate and can block antibiotics from reaching their targets on the bacterial cell (47). The concentration of antibiotics in urine can also increase their effectiveness, such as with daptomycin (10). Estrogen also influences the urogenital microbiome, which can lead to changed ecology and altered antimicrobial susceptibility (48). The presence of proteins and the ability of bacteria in specific environments to create them (such as beta-lactamases or disulfide bonds in the cell envelope) can directly determine individual antibiotic resistance and protection of other species by destroying antibiotics or blocking them from reaching their target. For example, disrupting protein homeostasis in cystic fibrosis-associated bacteria inactivates resistance mechanisms (49). Blood can contain antimicrobial peptides and other immune cell defenses that the bacteria must be able to defend themselves against (50).

The fluidics of environments, such as the bloodstream and urinary tract, is another issue bacteria must adapt to in order to infect. Bacteria must either adapt to flow with the environment or anchor themselves to prevent washing away. Certain bacteria, like *E. coli*, use pili to attach (51), while others use adhesions. In other environments, movement can be difficult, such as entering the brain through the blood–brain barrier. Bacteria must be able to interact with host cells and have signaling mechanisms in order to be able to cross and infect the brain (52). Media mimicking specific infection environments can help to account for some of these site-specific stressors and provide researchers and clinicians with more detailed information about antimicrobial susceptibilities. Currently, the standard medium for AST is CAMHB, which is a nutrient-rich medium without significant antimicrobial stressors. However, no infection sites exist that have high nutrient availability and no stressors, and therefore, using CAMHB to obtain AST results is not truly indicative of what is happening in infection environments. *P. aeruginosa* displayed increased tolerance toward colistin in cystic fibrosis sputum media as compared to the standard media. When media is supplemented with pyrimidines (especially uracil), *E. coli* shows increased susceptibility to antibiotics (53). When performed again *ex vivo*, the same tolerance as in the sputum media was shown (54). Bacteria can also be more susceptible to antimicrobials in different media as compared to the standard media, as the nutrients available directly influence AST results (55, 56).

It is well understood that the immune system plays a key role in host defense against pathogenic bacteria; however, recent research has shown there is still much more to be understood about how the host immune system interacts and synergizes with antimicrobials. Antibiotics can work directly with the immune system, upregulating production of important immune system defenses, while reducing the production of pro-inflammatory cytokines (57–59). Bacterial adaptation to immune system stressors can lead to decreased dissemination of the bacteria across the body. For example, methionine sulfoxide reductase mutants of *Staphylococcus aureus* were less likely to be killed by host oxidative stress but were also less likely to disseminate to the spleen (60). Understanding how to synergize antimicrobials with the host immune system is critical for effective antimicrobial prescription.

## POLYMICROBIAL INTERACTIONS IMPACT ANTIBIOTIC EFFECTIVENESS

Polymicrobial infections are a huge burden both on individuals and on the healthcare system (5–7). However, even knowing this, there is presently a lack of research on the effects polymicrobial communities have on the minimum inhibitory concentrations of individual organisms to different antibiotics, as most research focuses on identifying the resistance mechanisms of one causative agent (61). It has been shown that many infections are polymicrobial in nature (especially in diabetic foot ulcers, infections in immunocompromised individuals, and infections around implants or prosthetics) (62–64) and that the presence of multiple microorganisms can affect the growth responses and antimicrobial susceptibilities of individual organisms within their infection environments via polymicrobial synergism or antagonism (65–67). It has also been shown that bacterial communities can be specific to each body site and alter their community composition and distinct ecological niches during active infection. For example, *S. aureus* and *P. aeruginosa* often display antagonism toward each other; however, in the lung environment, where surfactants are common, they can work together to help colonize and infect (68). Bacteria adapting to their environment can also protect other microorganisms, such as *P. aeruginosa*, upregulating its biofilm formation during stressful conditions, which other organisms can benefit from to survive (35).

**Fig 2:**
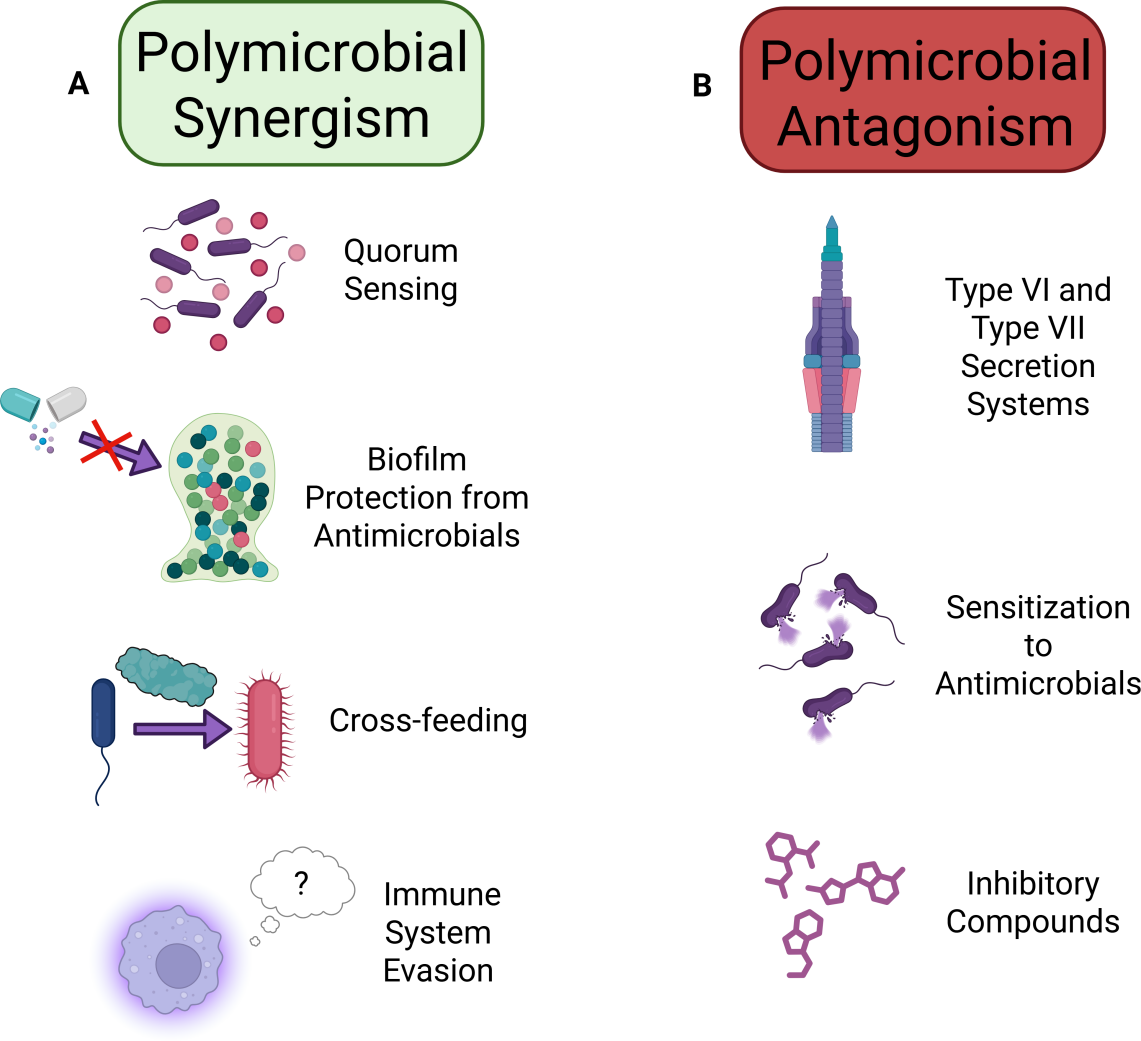
Mechanisms of polymicrobial synergism and antagonism. Microorganisms can synergize with one another (**A**) or antagonize one (**B**) via multiple mechanisms, including the ones described above. Synergism versus antagonism often depends on environmental conditions, including availability of resources, presence of stressors (such as the immune system or antibiotics), and the dynamics presented by other community members.

Polymicrobial synergism occurs when multiple organisms work together within the infection environment (Fig. 2A) and is an infamous cause of antibiotic recalcitrance (69–71). There are many mechanisms of polymicrobial synergy, including but not limited to quorum sensing, aiding in immune system evasion, cross-feeding, and protection from antimicrobials. Bacteria often place themselves in a structural hierarchy within their environment to allow for effective protection from stressors and sharing of resources (72). Organisms such as *P. aeruginosa* can enhance the colonization and persistence of organisms like *S. aureus* in lung infections (68), while other microorganisms can protect diverse members of their polymicrobial communities from various antibiotics. For example, *A. baumannii* can protect other bacterial species from antibiotics like meropenem (73), while *E. faecalis* can shield other microorganisms from aminoglycosides (74). The mechanisms of polymicrobial synergism can be directly mediated. For example, beta-lactamases produced by one bacterium can protect other bacteria from beta-lactams (75). However, other mechanisms of polymicrobial synergism may be indirect. For example, pyoverdine production and enterobactin production (siderophores) displace iron from cefiderocol, rendering the antibiotic useless as susceptible bacteria no longer take it up (76).

Polymicrobial synergism can also support the growth of organisms in different environments. *E. faecalis* takes advantage of damage done by *Clostridioides difficile* to acquire host heme, as it does not produce heme on its own. This heme acquisition allows *E. faecalis* to aerobically respire, altering its niche in the host environment (77). *E. coli* promotes *E. faecalis* growth in environments containing urine, and they are often found in catheter-associated urinary tract infections together (78). In return, *E. faecalis* helps *E. coli* prevail over iron restriction by the host (79). Polymicrobial community members can also provide alternative carbon sources in stressful environments, such as *S. aureus* providing acetoin to *A. baumannii* (80). Polymicrobial synergism can lead to antimicrobial recalcitrance through the sharing of resources, freeing up more energy for combatting the antibiotic or other stressors.

Members of polymicrobial communities can also work together against the immune system. *P. aeruginosa* can produce multiple compounds that increase other organisms’ oxidative stress tolerance, including pyocyanin (which does this directly for *A. baumannii*) (81) and 2-heptyl-4-hydroxyquinoline N-oxide (HQNO) (which induces staphyloxanthin (STX) in *S. aureus*, leading to resistance toward hydrogen peroxide) (82). *E. faecalis* can protect *S. aureus* from NETosis (release of neutrophil extracellular traps), leading to increased survival for both organisms (83). *S. aureus*, *P. aeruginosa*, *E. faecalis*, and *A. baumannii* all respond to exogenous peptidoglycan by forming biofilms, which can prevent both the immune system and antimicrobial penetrance (84).

There is often a fine line between polymicrobial synergism and antagonism, with many bacteria switching between the two depending on environmental context. *P. aeruginosa* can antagonize *E. faecalis* but will attenuate competitive behavior depending on their proximity to each other (85). An organism that may cooperate with one organism (such as *P. aeruginosa* with *Enterococcus faecium*) may compete against a different organism (*P. aeruginosa* with *E. coli*) even within the same environment (86). By accounting for polymicrobial synergism when determining AST, we can prescribe antimicrobials more effectively (87). By disrupting synergy mechanisms, such as quorum sensing, we can decrease virulence and improve patient outcomes (88).

Polymicrobial antagonism, the phenomenon in which one or more organisms act in opposition toward another (Fig. 2B), can also alter antimicrobial susceptibilities. *P. aeruginosa* increases the susceptibility of *S. aureus* to antimicrobials that target its membrane by producing HQNO, which increases its membrane fluidity (89), and LasA produced by *P. aeruginosa* allows vancomycin to kill *S. aureus* more easily (90). Organisms can also inhibit the growth of other organisms in nutrient-depleted environments, such as the inhibition of *P. aeruginosa* growth by *E. faecalis* in iron-restricted environments (91). Production of inhibitory compounds, such as the production of phenazine-1-carboxamide by *P. aeruginosa* to antagonize *A. baumannii*, can also decrease the growth of other organisms (92). However, in response, the organisms can no longer evolve to be inhibited by these molecules or to outcompete their competitor (93).

Polymicrobial antagonism can be through secretory compounds like phenazine-1-carboxamide or the upregulation of pyocyanin production through polyphosphate in *P. aeruginosa*, or through secretion systems like the Type VI or Type VII secretion systems (94). Even if one bacterium is not directly competing against the other, some bacteria will still attack bystanders, such as *E. faecalis*, using its Type VII secretion against bystander *S. aureus* (95). By exploiting bacterial competition, we can drive increases in susceptibility to antimicrobials, allowing for the more effective clearing of polymicrobial infections.

## CONCLUSIONS: IMPROVED AST SHOULD ACCOUNT FOR ENVIRONMENT AND BACTERIAL COMMUNITIES

Antimicrobials face many challenges as they travel through the body to the infection site to do their jobs and be effective. They must have the proper pharmacokinetics to make it to the appropriate body site, the correct pharmacodynamics to do their job once they are at that body site, and must be able to effectively target bacteria that have adapted and evolved to their site-specific stressors and polymicrobial communities when present. To improve antimicrobial prescription and promote good antimicrobial stewardship, our AST methods must improve. Recently, there have been many improvements promoted in the research setting, with a few making it to the clinical setting, including rapid diagnostic methods like sequencing and microfluidic-based optical and electrochemical AST that accounts for metabolic activity and electrochemical signals produced by the bacteria during antibiotic exposure (96).

There are those arguing to skip culture-based methods altogether and to instead focus on the more rapid method of using next-generation sequencing and other sequencing technologies to predict AST results without ever having to culture the bacteria (97). Some scientists argue that improving upon culture-based methods using media more indicative of the nutrient composition found in different infection environments is enough to more accurately gauge antimicrobial susceptibilities (for example, using RPMI 1640 or a nutrient-depleted medium, unlike the standard CAMHB, which is considered nutrient-rich) (98, 99). Studies have shown that nutrients available in media can influence bacterial activity, such as siderophore production in media that is iron-rich or iron-limited (100). Nutrient availability can also impact antimicrobial susceptibility, such as *P. aeruginosa* displaying increased susceptibility to tobramycin in media containing glucose but decreased susceptibility in media containing lactate (101). These studies, as well as others, attempt to address the issues of performing AST in standard media as compared to media that mimic environmental conditions at different infection sites.

There is also the keystone-pathogen hypothesis, which states that due to polymicrobial interactions within an infection environment, targeted disruption of a critical species could cause community collapse, thus clearing the infection (102, 103). Accounting for polymicrobial synergism and antagonism has the potential to help determine AST results more accurately as compared to performing AST with a monomicrobial culture (87). By fully accounting for the bioburden of infections (the size and mass of the infection as well as the biofilms potentially produced by the bacteria within the infection), we can more effectively prescribe antimicrobials and improve patient outcomes (102). By understanding the pharmacokinetics and pharmacodynamics of antimicrobials relevant to infection sites using relevant models, we can make sure that we not only prescribe antimicrobials that will work *in vitro* but also in the patient (23).

**Fig 3:**
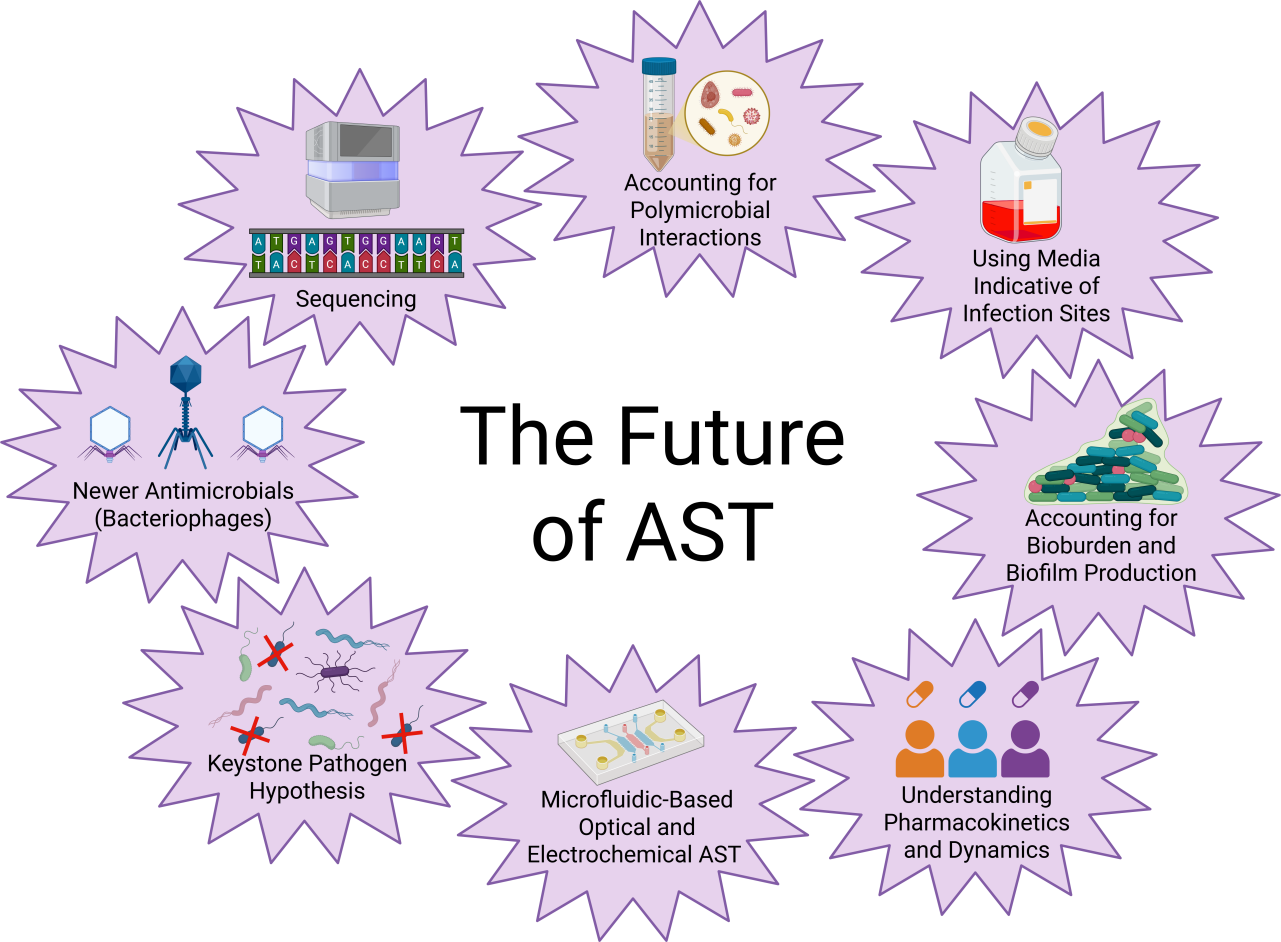
Strategies for improving AST and antibiotic prescription for the future. Improving AST and antibiotic prescription in the future will take a combination of multiple strategies like the ones listed above.

As we look to the future of AST, we also look to the future of combatting antimicrobial resistance. Bacteriophages have become an alternative to antibiotics, prescribed for compassionate use in highly resistant infections, and the synergy between bacteriophages and antibiotics, and studying how they can work together, may open many doors for treating resistant infections in the future (104, 105). While all the above methods for improving AST and combating antimicrobial resistance hold promise, the best improvements to AST and antibiotic prescription are likely a combination of methodologies (Fig. 3). There are many challenges to prescribing antibiotics for different pathogens adapted to unique body sites and to determine how to overcome them will likely take more than one AST strategy. The overarching goal of AST should consider not only the pathogen but also its niche and how it has adapted to its environment and the other microorganisms around it.
